# Brain Point-of-Care Ultrasound (BrainPOCUS) in Adult Critical Care: An Introduction to Methods

**DOI:** 10.7759/cureus.100009

**Published:** 2025-12-24

**Authors:** Richard M Cashmore, Avinash K Jha

**Affiliations:** 1 Critical Care, Royal Papworth Hospital, Cambridge, GBR; 2 Critical Care, Royal Preston Hospital, Preston, GBR

**Keywords:** brainpocus, critical care, critical care ultrasonography, intensive care, neuropocus, neurosonology, point-of-care ultrasound (pocus), transcranial doppler ultrasonography, ultrasound in critical care

## Abstract

Brain point-of-care ultrasound (BrainPOCUS) is a relatively recent addition to the critical care clinicians' POCUS armamentarium. Whilst the transcranial ultrasound techniques employed have origins dating back to the 1950s, it is only more recently that the potential role of a POCUS brain examination in critical care settings has gained more widespread general interest and recognition. The information BrainPOCUS can provide in critically ill sedated or comatose patients is unique and informs about pathophysiology typically undecipherable from physical examination alone. BrainPOCUS in critical care is an evolving technique, finding its place somewhere between a basic bedside physical examination and a classical comprehensive neurosonology investigation. This article aims to provide the BrainPOCUS novice with a step-by-step guide to practical aspects of B-mode imaging, the transcranial colour-coded duplex (TCCD) technique, and the transcranial colour-coded triplex (TCCT) technique. Intended as a methodological introduction for an intensivist. Thus, further study, practical training, data synthesis and understanding, and formal accreditation pathway completion are required.

## Introduction

Background

Neurological or brain ultrasound is believed to stem from the 1940s, at which time 'hyperphonograms' were studied before being discounted due to bony artifact from the skull [1]. In the 1950s, using a 'modified flaw-detector', transcranial pulsed frequency echoes were successfully shown to demonstrate the presence of certain midline structures from an intact skull [2]. Such a device reportedly went on to guide the identification and surgical management of an intracranial haematoma in a paediatric patient, likely representing the first clinical application of brain point-of-care ultrasound (BrainPOCUS) [1,3]. In the 1980s, Aaslid et al. published their landmark paper on Doppler ultrasound examination of the basal cerebral arteries, demonstrating the ability of a Doppler waveform to be obtained from the middle cerebral artery (MCA), insonated through the relatively thin temporal bone of the skull [4]. From this point, brain ultrasound has been progressively explored with subsequent advances in physiological understanding alongside technological developments [5]. Despite this, it is only more recently that the potential utility of a constrained and rapid BrainPOCUS exam in the contemporary management of critically ill adults has again been recognised. With critically ill patients frequently sedated or comatose, and bedside clinical examination findings accordingly limited, the information provided through bedside BrainPOCUS is particularly valuable.

Introduction

BrainPOCUS combines transcranial brightness-mode imaging, colour Doppler, and pulse-wave Doppler to deliver a rapid structural and haemodynamic assessment at the bedside. BrainPOCUS is deliberately focused, aiming to rapidly provide basic but crucial information in time-critical situations. BrainPOCUS should not be confused with a complete diagnostic neurosonology examination, the specialist area of which and associated practice standards are well defined [6]. Within critical care, consensus recommendations on brain ultrasonography have defined the basic skill set needed [7]. This article, written as a methodological guide, covers only the 'basic' and some 'basic plus' practical performance aspects [7]. The ability of BrainPOCUS to inform the clinician at the bedside of life-threatening intracranial pathology, along with the real-time cerebrovascular status of the patient are of particular value to intensivists, their patient population, and the physiological support techniques employed. BrainPOCUS is not a substitute for the comprehensive and pathway-linked investigations such as computed tomography (CT) and magnetic resonance imaging (MRI); however, it can provide valuable supplementary and complementary information. BrainPOCUS offers significant advantages through immediate bedside availability in patients for whom transfer poses additional risk, repeatability without exposure to ionising radiation, and provision of highly dynamic physiological data in the precise physiological context. Earlier BrainPOCUS limitations have included operator dependence and variation, which, through technological advances and standardised approaches such as those selected here, can be increasingly mitigated.

Potential applications

The potential applications of BrainPOCUS are ever-increasing; however, the field remains principally limited by the lack of large-scale population data and interventional studies to date [5]. BrainPOCUS can be used to help identify a number of acute pathologies, including midline shift, hyperaemia/vasospasm, raised intracranial pressure, and impaired cerebral perfusion. Fundamentally, BrainPOCUS provides real-time data on cerebrovascular haemodynamic status and basic structural appearance. IMPRESSIT-2, a particularly notable study in the field, demonstrated the possible role of transcranial Doppler (TCD) in ruling out intracranial hypertension (noting its high negative predictive value) in acute brain-injured patients [8]. The B-ICONIC consensus published in 2025, for the use of non-invasive intracranial pressure monitoring in patients with traumatic brain injury where invasive systems are not available, further highlighted the potential role for BrainPOCUS [9]. Application of BrainPOCUS in more novel settings and settings that currently have less of a primary neurological focus are expected to increase over the next decade, but currently have even less application and evidence [5]. Broader application of BrainPOCUS through robust and ethically approved studies may help further pathophysiological understanding of the neurological sequelae in critically ill disease states and could help tailor intensive care physiological supports.

## Technical report

Physics/Safety

Ultrasound is a high-frequency mechanical oscillation. It uses frequencies higher than those of human hearing and, importantly for medical imaging, can be formed into narrow beams that can be transmitted and received [10]. The mechanical oscillation itself is important to consider due to thermal and mechanical stresses applied to the tissues being examined. An approach of 'as low as reasonably achievable' (ALARA) should always be followed when imaging biological structures, with specific additional care and precautions required for optic nerve sheath diameter assessment, as outlined in previous studies [6,11].

The Doppler principle is used to assess changing velocity in relation to ultrasound waves reflecting from a moving object, in this case, blood. Whilst it is very easy to consider transcranial Doppler waveforms as 'flow' (confounded by classical terms such as 'flow velocity'), it is important to remember that it is velocity only (speed and direction) that is measured by Doppler. To measure flow itself, an area measure to facilitate volumetric assessment along with the velocity data is required, and additional rheological factors require further consideration.

Positioning/ICU modifications

**Figure 1:**
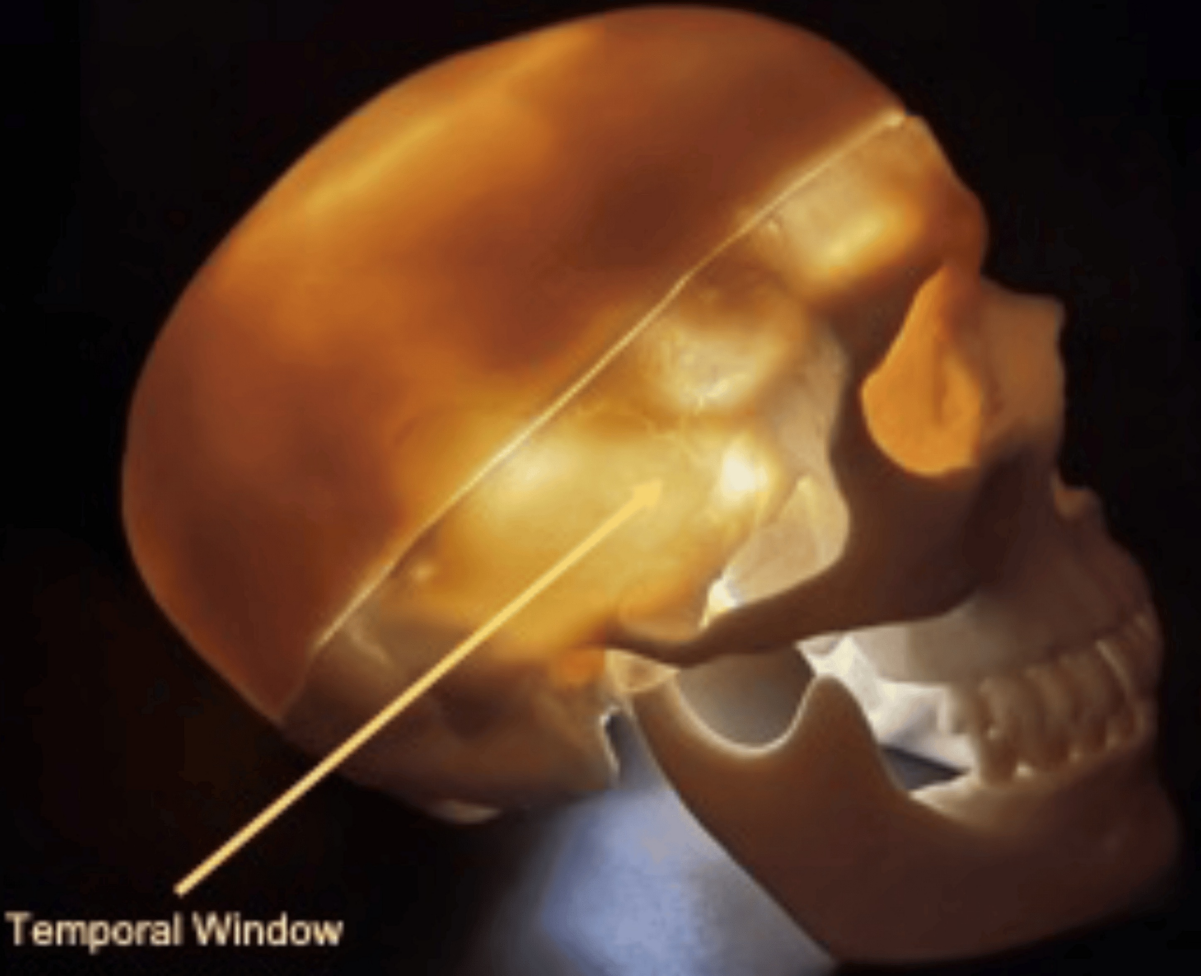
A plastic skull model used to demonstrate the relatively thin temporal bone window used for BrainPOCUS. BrainPOCUS: Brain point-of-care ultrasound

With the brain surrounded by the bony cranial vault, only a limited number of probe positions can allow ultrasound transmission and sonographic examination. These positions are referred to as 'windows', allowing various views of the intracranial vault. As Aaslid et al. demonstrated, the temporal bones bilaterally are relatively thin structures well suited to facilitate ultrasound passage [4] (see Figure 1). The bilateral temporal windows remain the transcranial windows used principally during a basic BrainPOCUS exam, including for assessment of intracranial midline and Doppler analysis of the middle cerebral arteries [12]. 

The temporal window is located above the zygomatic arch, with the probe placed in a horizontal plane in line with the orbit. The probe marker is directed towards the eye (reversed in cardiac presets). Of note is that approximately 10-20% of the population (in particular, those of increased age) can lack transtemporal window adequacy [13]. To initially confirm window presence, it is advised to set the insonation depth to in excess of 15 cm. With this, the sonographer can look for the highly acoustically reflective inner table of the contralateral skull to confirm that a basic window is present. Following this confirmation, depth should be reduced to initially align the contralateral temporal bone table with the lower border of the screen. It is worth noting that, even with a confirmed sonographic window, though visualisation of the contralateral temporal bone, variable degrees of window quality can be seen.

**Figure 2:**
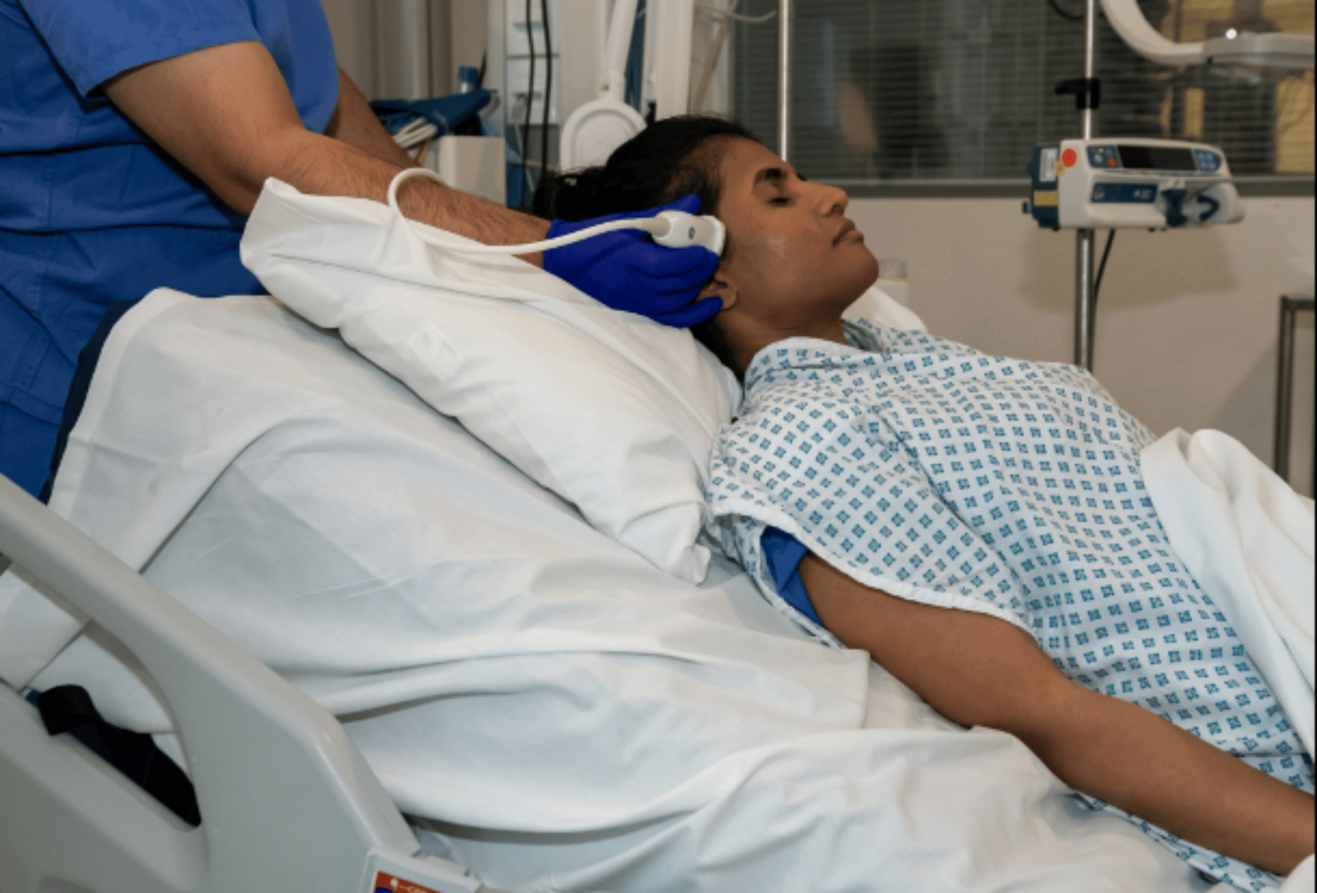
A simulated demonstration of the positioning for BrainPOCUS in the critical care unit. It is not infrequent that bedside access itself is challenging and limited, with scanning occasionally required from alongside the patient. It is helpful to rest your hand on a pillow and gently against the patient for the fine motor adjustments required. BrainPOCUS: Brain point-of-care ultrasound

Optimal patient positioning is infrequently possible in the critically ill cohort, either due to an emergency setting or the presence of attached supportive therapeutic equipment. Typically, BrainPOCUS is performed with the sonologist and ultrasound device positioned behind the patient's head (see Figure 2), but can be performed from the lateral position as required. In the setting of trauma, it is of utmost importance to consider and maintain appropriate spinal precautions. The presence of a cervical spinal collar/immobilization can prevent submandibular window access.

**Figure 3:**
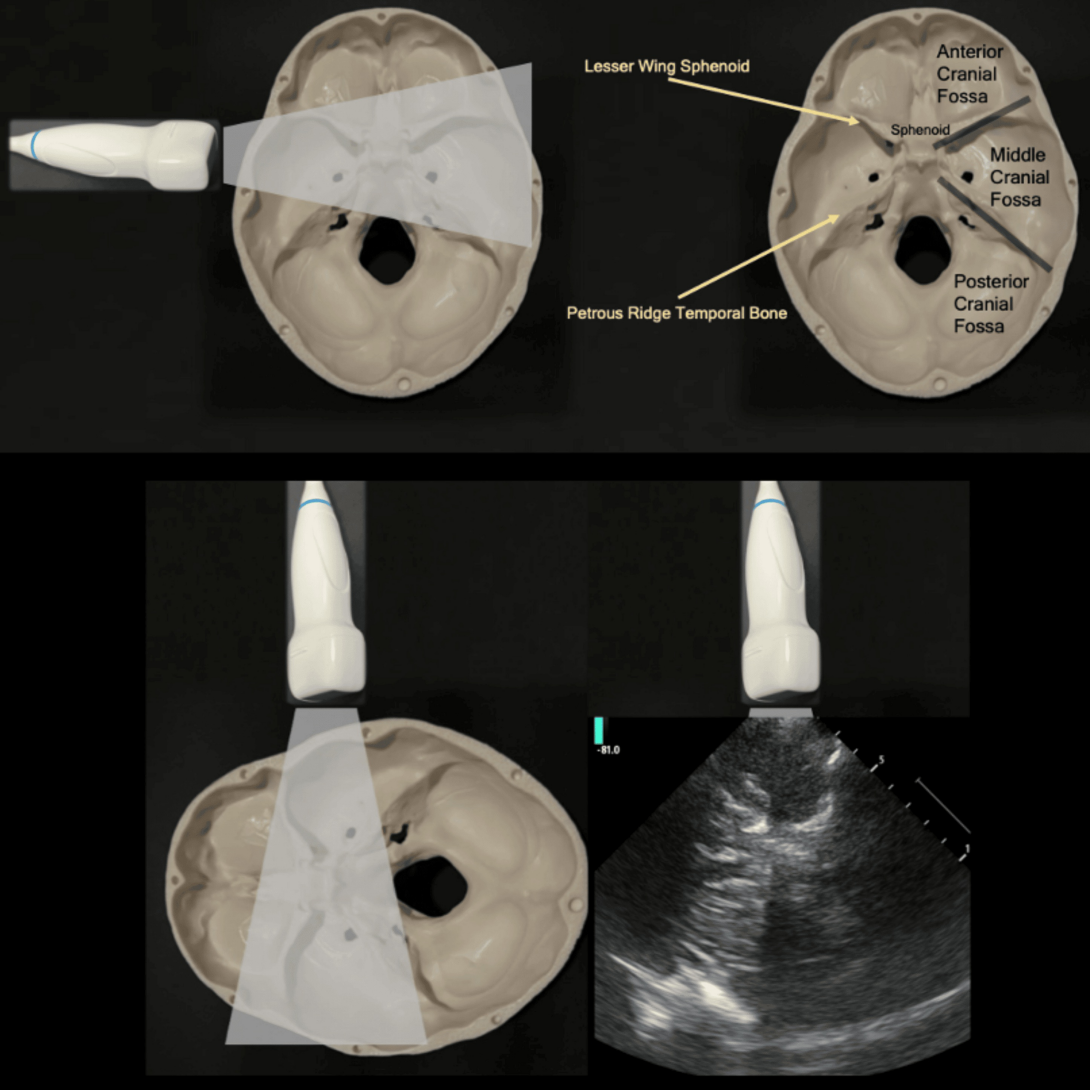
The transtemporal window. The upper panes display the relevant bony anatomy in the axial plane. The lower panes display an orientation example of right temporal window axial insonation.

Transtemporal window: Bony landmarks provide a fundamental base from which window adequacy assessment, B-mode orientation, and navigation can be performed (see Figure 3). The brain can be assessed in multiple planes through multiple sonographic acoustic windows. For the purposes of brain imaging in BrainPOCUS, the transtemporal window and axial plane alone are used, though the probe footprint can be moved into more anterior/posterior/superior/inferior regions over the temporal bone depending upon individual patient window characteristics. Of note, the transtemporal window in the axial plane produces B-mode images similar in appearance to those of a typical axial CT scan.

**Figure 4:**
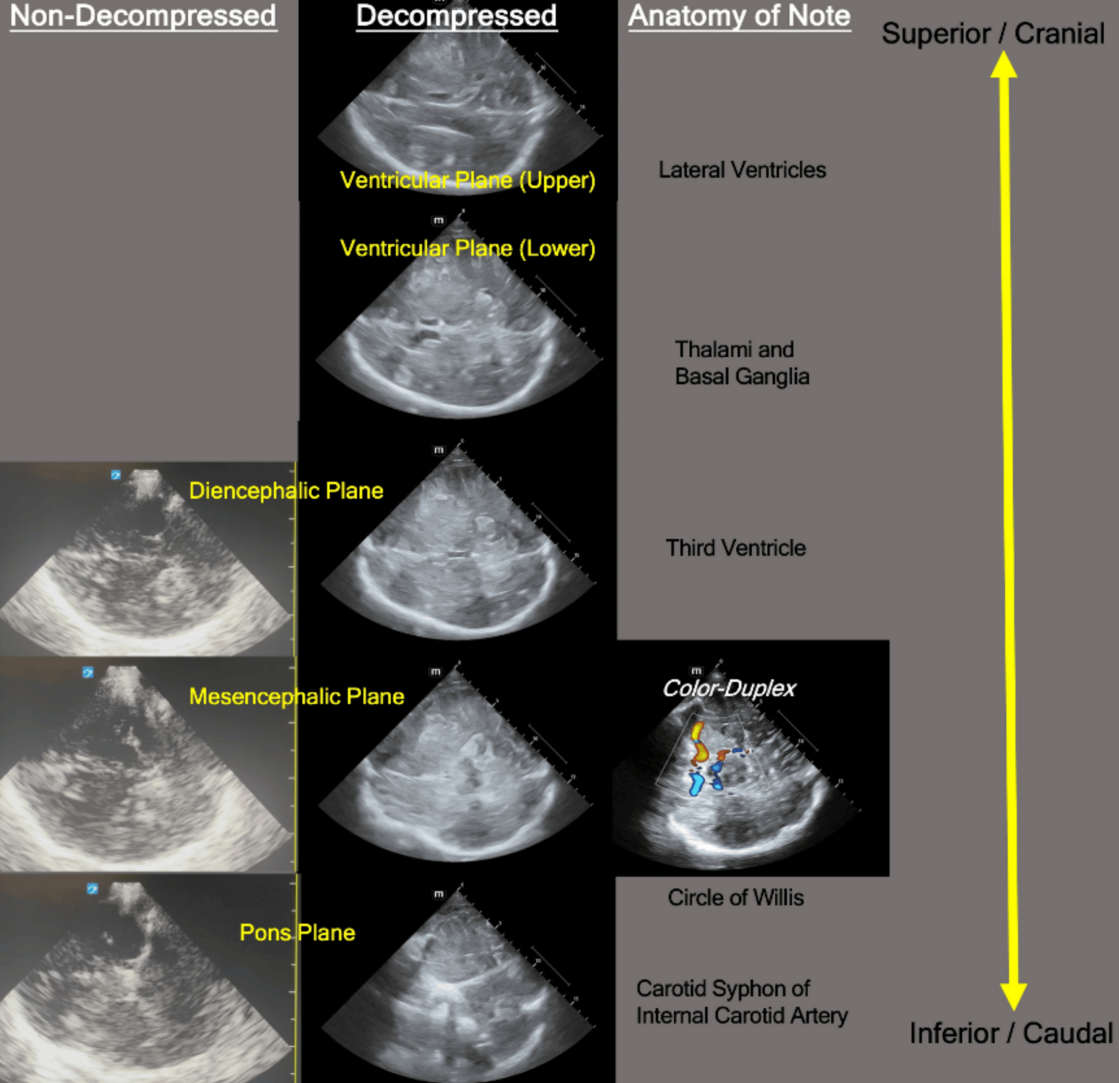
Transcranial B-mode imaging ultrasound in the axial planes via the transtemporal window. The left column displays images from a non-decompressed individual. The middle column displays images obtainable in a patient following decompressive craniectomy. Of note, in the above decompressed example, there is mass effect and ventricular distortion. The right column has additional colour-duplex imaging to highlight the location of the Circle of Willis anterior to the midbrain.

The temporal window in the axial plane is then further examined in a number of B-mode 'horizontal' (technically off-axis diagonal) planes by tilting the tail of the probe up-and-down. Each of these subsequent planes is named according to its structural content origin (see Figure 4) [14]. Importantly, these axial planes lack hard borders per se and can at times be seen overlapping within a single image, particularly if a small degree of probe rotation has occurred. Appreciation of these planes is useful for structural orientation and navigation, along with assessment and later vessel identification.

Structural/B-mode landmark navigation: With the probe placed over the temporal bone, and B-mode imaging in the axial plane, begin by bringing the tail of the probe superiorly and imaging the base of the skull. Several bony structures should be visible, with two principal diagonal lines seen converging towards the middle of the cranial vault (sphenoid), before diverging distally past the midline towards the contralateral temporal bone (see Figure 3). These diagonal echogenic bony structures are the lesser wing of the sphenoid (anteriorly) and the petrous ridge of the temporal bone (posteriorly).

Whilst embryological nomenclature appears widely in the literature, what is most relevant to the BrainPOCUS sonographer is the B-mode identifiable midbrain (butterfly-appearing) structure (see Figure 4). The midbrain, imaged in the axial plane at the mesencephalic level, is formed from the cerebral peduncles, appearing butterfly-like with the 'wing-tips' facing anteriorly. The midbrain itself is well demarcated on sonographic exam due to the cerebrospinal fluid-parenchyma interface with the basal cisterns. Identification of the mesencephalic plane is important as it is the level at which the basal intracranial vessels' primary divisions and the Circle of Willis are found.

By continuing to tilt the probe tail down and image more superiorly, the axial diencephalic plane should come into view. The diencephalic plane is at the level of the third ventricle, itself appearing as two well-demarcated echogenic parallel lines, usually in the center of the cranial vault. This anatomically midline echogenic structure can be used to assess for midline shift. Care needs to be taken as there can be artifactual lines with a similar appearance.

**Figure 5:**
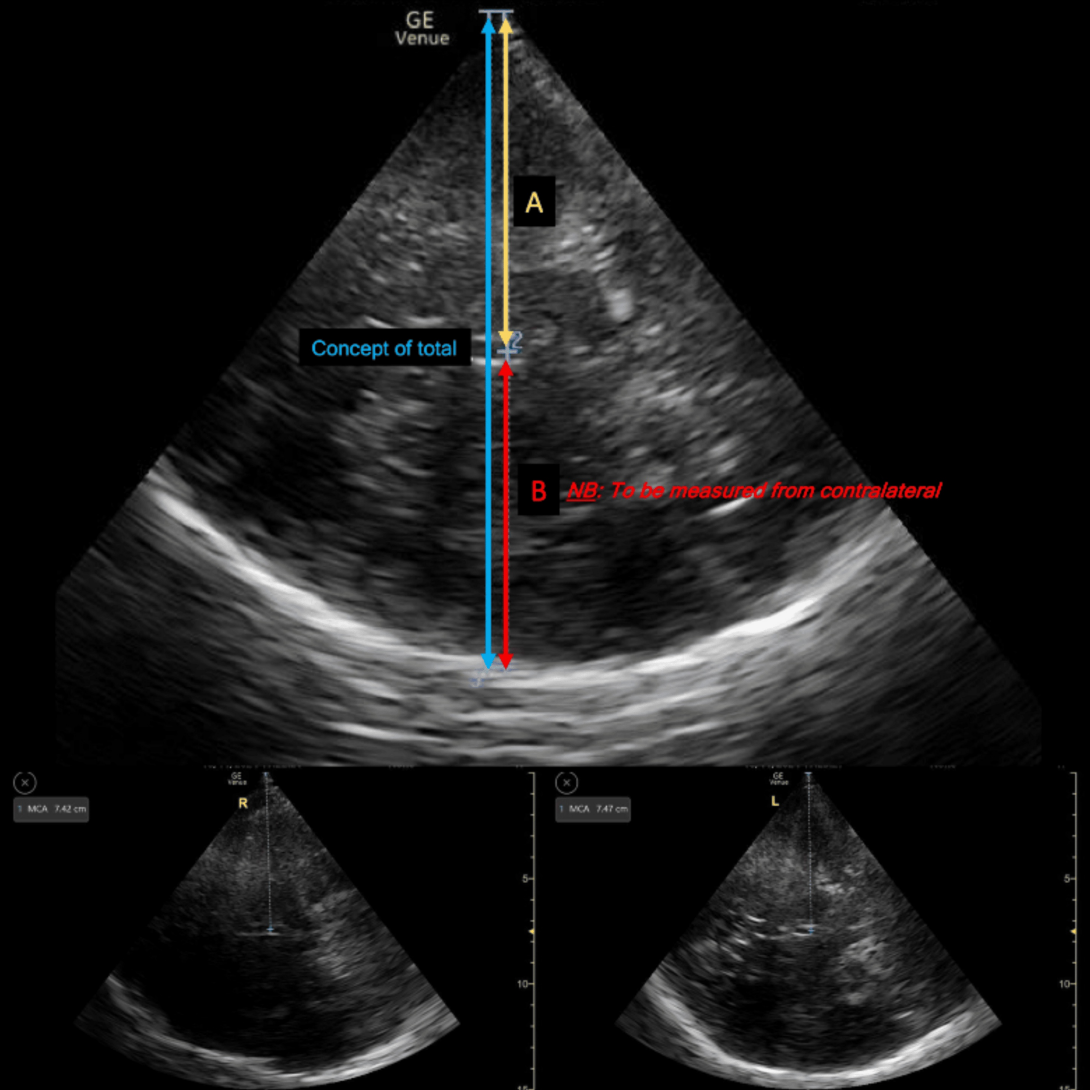
Displacement of the third ventricle to assess for the midline shift. The below two images use depth assessments from both right and left temporal windows. They are used as the inner table of the ipsilateral temporal bone is not clearly visible and the probe can overestimate ipsilateral depth if attempts to measure are made from one side only. The uppermost image is used to demonstrate the theoretical total line being measured in two parts from each ipsilateral window.

Despite its historical origin, assessing midline shift can at times still be challenging, and care is required to avoid artifact misinterpretation. With the third ventricle imaged, and importantly, B-mode image positioning and angulation such that depth measurement is in a line at 90 degrees (right-angle) to the ventricular margins, the distance between the ultrasound probe and the centre of the third ventricle can be measured [15]. This measure is repeated on both sides of the head, and the formula (Ipsilateral Depth - Contralateral Depth) / 2 is used to calculate relative dislocation or shift of the third ventricle (see Figure 5).

$\text{Dislocation of 3rd Ventricle (Midline Shift)}=\frac{\left( A-B \right)}{2}$

**Figure 6:**
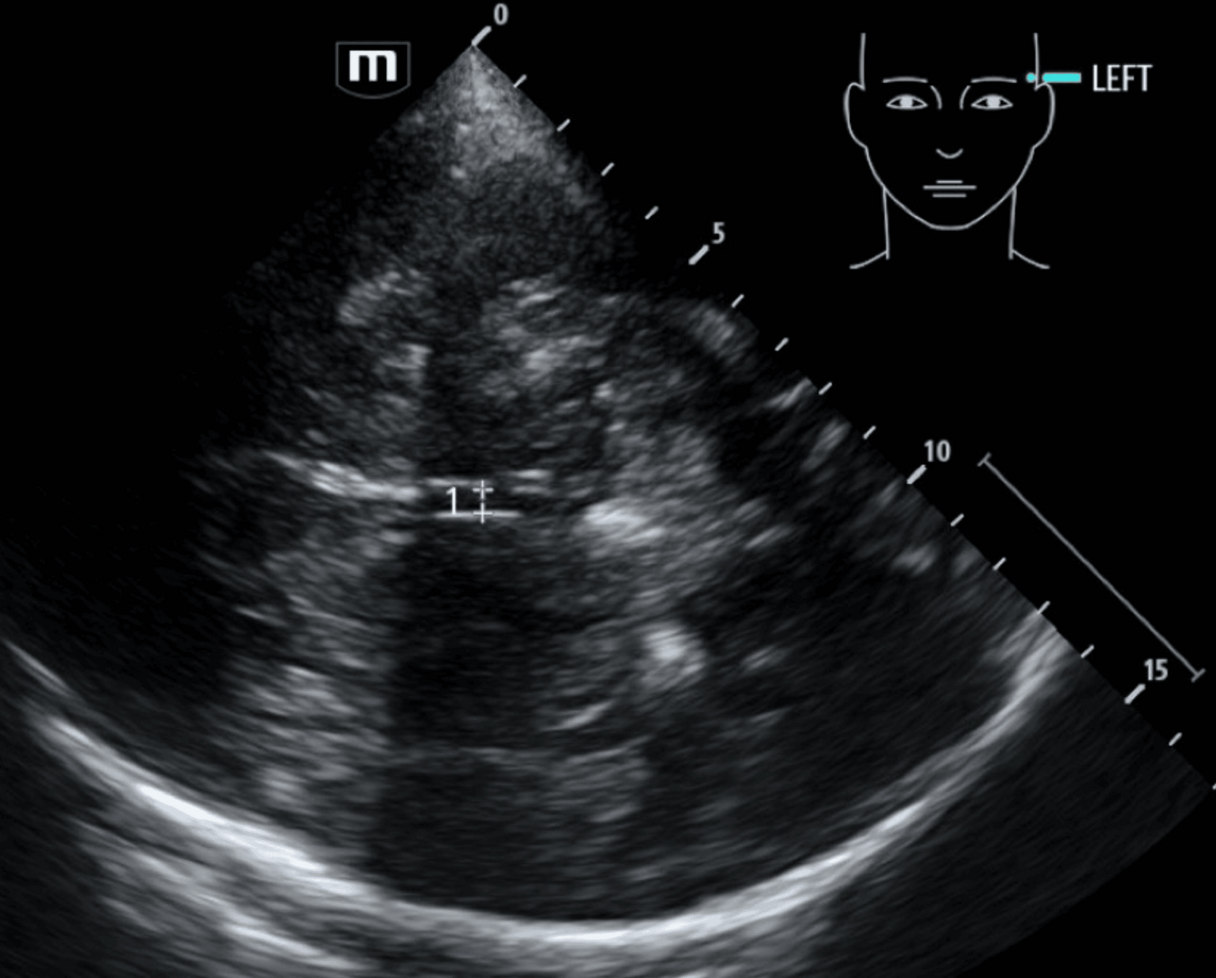
Temporal B-mode image of the third ventricle, appearing as two echogenic parallel lines in the midline.

Relatively simply, the diameter of the third ventricle can also be measured from the diencephalic plane. Whilst cut-off values have been sought [16], it is perhaps currently best suited (as many aspects of structural BrainPOCUS) to serial assessment in-between comprehensive CT to assess for interval changes (see Figure 6).

Interrogation of MCA: The bilateral MCAs supply a large volume of brain tissue and may display the effects of intracranial flow/pressure change. Importantly for BrainPOCUS, the MCAs are relatively easy to identify on transcranial colour-coded duplex (TCCD). There are notable limitations in regard to isolated MCA assessment, including a lack of any posterior circulation assessment or any comprehensive neurovascular assessment, but it can serve as a good starting point for basic BrainPOCUS [7]. TCCD examination of the MCA has been recommended as a basic skill for the intensivist to rule out intracranial hypertension impairing cerebral perfusion [17], and protocols for the TCCD assessment of the MCA alone have been published for some specific indications [12].

**Figure 7:**
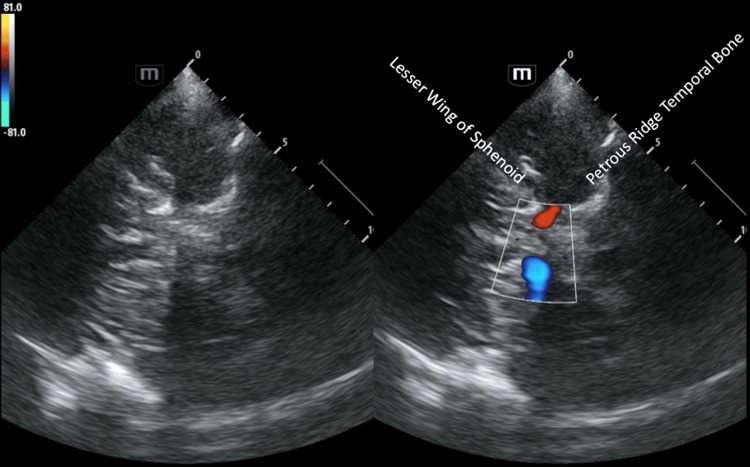
Demonstration of a transcranial B-mode (left) and colour-coded duplex image (right) of the base of the skull, highlighting the bilateral carotid siphons.

When starting, it is often useful to orientate with caudal 'hard' bony landmarks due to their bright echogenicity (see Figures 3-4). From this, begin tilting the probe tail down slowly to progressively move the field of insonation more cranially until the mesencephalic plane (indicated by the presence of the butterfly of the midbrain) is identified. If struggling to identify the midbrain on B-mode, a colour box applied over the sphenoid can help locate the distal carotid siphon, which can then also be followed superiorly along its course using TCCD to the MCA (see Figure 7).

With the mesencephalic plane identified, place a colour-doppler box anterior to the cerebral peduncles, corresponding to the anatomical location of the Circle of Willis. The ipsilateral MCA should appear prominent (see Figure 4) with the vessel projecting towards the probe (red in colour with default Doppler colour settings) at an off-vertical angle initially. With some subtle adjustment (often slight fanning and footprint adjustment), the MCA should be brought towards the middle of the screen for assessment. By tilting the tail of the probe up and down in a cranio-caudal fashion, the MCA segments can be explored in further detail. With the ipsilateral MCA identified, the scanning depth can be reduced to allow improved resolution and more detailed pulse-wave gate placement, though this is not always required for BrainPOCUS. An appropriate colour scale pulse-repetition frequency should be used to avoid aliasing.

**Figure 8:**
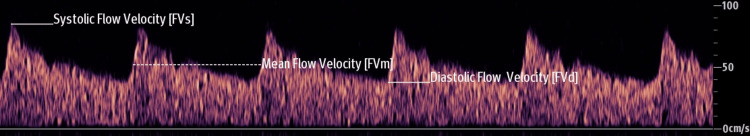
A typical normal low-resistance middle cerebral artery (MCA) Doppler waveform.

Pulse-wave Doppler (PWD): The pulse-wave sample gate should be placed over the TCCD-identified MCA M1 region, with MCA M1 midpoint at approximately 50 mm depth (range: 45-55 mm) [6]. The default PWD gate size is usually 5 mm [18] and again is probably reasonable without adjustment for the purposes of BrainPOCUS. Angle correction can be applied when adequate vessel lengths are visible on duplex to ascertain the trajectory, but its use has been debated. In health, the ipsilateral MCA should have a positive waveform, as blood is moving towards the probe in the transtemporal window with a low-resistance appearance (see Figure 8).

Applying the PWD gate sequentially along the TCCD-identified vessel course can facilitate a more comprehensive Doppler assessment of the vessel (e.g. focal pathology), but is beyond the remit of basic BrainPOCUS. As is often the case in medicine, where asymmetrical findings occur, concern should be for more focal pathology, and further urgent expert examination/comprehensive imaging modality should be undertaken as appropriate. Using both the initial colour-duplex vessel appearance and spectral Doppler waveform appearance, some basic inferences can be rapidly made (e.g. diastolic flow adequacy or flow reversal), though typically more objective numerical measurements are required.

**Figure 9:**
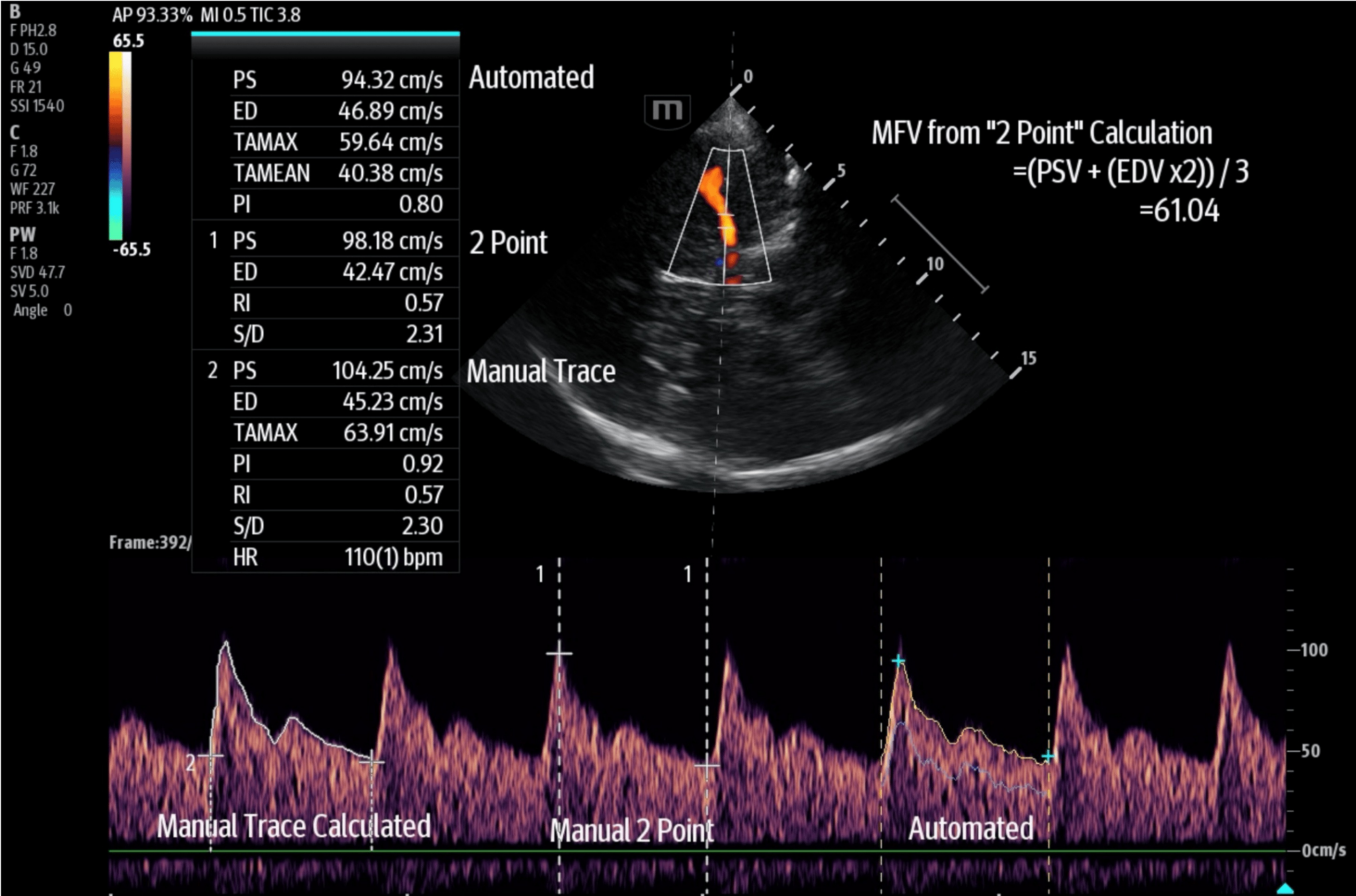
A demonstrative transcranial colour-coded triplex (TCCT) image of an MCA during BrainPOCUS, with various mean flow velocity calculation methods. Mean flow velocity (MFV) can be calculated or measured directly using time averaging of the maximal envelope follower. Automated TAMAX = 59.64 cm/s; manual trace TAMAX = 63.91 cm/s; two-point calculated MFV can be completed using the formula MFV=(PSV+(EDVx2))/3, which in above = (98.18+(42.47x2))/3=61.04 cm/s. As seen in this automated example, sometimes the true peak/waveform is not tracked precisely. Importantly, none of these values is to be confused with TAMEAN, shown in a light blue line within the Doppler trace in the automated section, which does not follow the maximal envelope. BrainPOCUS: Brain point-of-care ultrasound; MCA: middle cerebral artery

Within the Doppler waveform, the maximal frequency Doppler envelope describes the uppermost maximal velocity over the waveform cycle and is most frequently used for the purposes of measurement and calculation. ‘Time-averaged’ maximum velocity (TAMAX) and mean flow velocity (MFV) have been used interchangeably, with MFV manually calculated as (FVs + (FVd x 2)) / 3 [12] (see Figure 9). The authors here prefer the maximal envelope follower (or time-averaged maximum) over manual two-point calculation as global haemodynamic variables could alter portions relating to systolic and diastolic periods and are, in theory, more accurate. Evolution in technology, however, can make direct comparisons with historical data and associated techniques challenging at times. Various auto-calculation functions can achieve ‘outline’ measurements rapidly (though they can be readily erroneous, and much care is required to check returned values and waveform sampling). Manual trace of the uppermost Doppler signal edge for a single beat (or multiple beats to average in the setting of arrhythmia) can be performed and measured on the device. We suggest values both with and without angle correction are obtained, where possible, to allow more comprehensive comparisons if the net vessel trajectory is clearly visible for a suitable length.

At this point, it is critical to note key systemic variables. Cardiovascular changes such as cardiac arrhythmia may be obvious from the spectral Doppler waveform, but hypotension, or respiratory and haematological factors that can affect readings may be much less apparent without active investigation. Parameters at the time of examination require a concurrent record to correctly inform of the physiological status. An example BrainPOCUS parameter report has been included (see Appendix 1).

Normal values/morphology (MCA only): The intracranial arterial system is, in health, a ‘low-resistance’ vascular bed, and accordingly waveforms differ from the typical ‘high-resistance’ waveforms seen in the peripheral vasculature. Defining normality has been attempted by a number of authors (see Table 1), though it itself requires additional consideration given technological changes over time. It is important to consider systemic, local, and technical aspects when obtaining values, with repeated assessment affording the ability to track changes over time.

**Table 1:** Population-recorded norms for the middle cerebral artery Doppler assessment. Please note that Refs [19,20] used transcranial colour-coded duplex (TCCD) similar to BrainPOCUS, whilst Refs [6,21] involve non-Imaging transcranial Doppler (TCD). BrainPOCUS: Brain point-of-care ultrasound; MCA: middle cerebral artery

Studies	MCA FV _Systolic _(Cm/second)	MCA FV _Mean _(Cm/second)	MCA FV _Diastolic _(Cm/second)
Martin et al. 1994 [19], TCCD, Mean (±95% CI)	Age 20-39 years: 113 (109-116)	Age 20-39 years: 74 (71-76)	Age 20-39 years: 51 (49-53)
	Age 40-59 years: 106 (101-111)	Age 40-59 years: 72 (69-76)	Age 40-59 years: 47 (45-50)
	Age >60 years: 92 (88-96)	Age >60 years: 58 (55-61)	Age >60 years: 35 (33-37)
Krejza et al. 1999 [20], TCCD, Mean (± 2SD)	Age 20-40 years: 120 (64-176)	Age 20-40 years: 81 (41-121)	Age 20-40 years: 55 (29-81)
	Age 41-61 years: 109 (65-175)	Age 41-61 years: 73 (35-111)	Age 41-61 years: 49 (23-75)
	Age >60 years: 92 (58-126)	Age >60 years: 59 (37-81))	Age >60 years: 37 (21-53)
	All: 110 (54-166)	All: 73 (33-133)	All: 49 (21-77)
Tegeler et al. 2013 [21], Non-imaging TCD, Mean (± SD)	-	Proximal MCA: 60.1 (SD 12.1)	-
	-	Distal MCA: 60.6 (SD 12.5)	-
Alexandrov et al. 2007 [6], Non-imaging TCD, Mean	-	Practice Standard Paper Adult Mean Flow Velocity at Assumed Zero Degree Angle of Insonation: M1 MCA FV _Mean_: 30-80	-

PWD derivations: Pulsatility index (PI) is a dimensionless number constructed by Gosling et al., originally on the peripheral vasculature, to describe Doppler waveform morphology [22]. By its nature as a calculated ratio, PI is angle-independent [23].

$\text{Pulsatility Index (PI)}=\frac{\left( FV_{Systolic} - FV_{Diastolic}\right)}{FV_{Mean}}$

Later applied to transcranial Doppler waveforms, PI was seen initially to positively correlate with intracranial pressure in neurosurgical patients [24]. This observed correlation may be seen with isolated ICP rises in settings where other physiological parameters remain static. However, it is vitally important to note that rising ICP is not the only cause of a rise in PI, with some of the limitations and complexities well described in critical care [25] and potential for focal pathology. Work has been done to try to estimate CPP by incorporating non-invasive TCD values and mean arterial blood pressure (MABP) as a variable [26]. The formula noted importantly was derived from a specific head-injury cohort and is not validated in all ICU populations.

$\text{Non Invasive Cerebral Perfusion Pressure (nCPP)}=\text{MABP}\left( \frac{FV_{Diastolic}}{FV_{Mean}} \right)+14$

B-ICONIC, with its specific remit and traumatic brain injury cohort, recommended that for TCD/TCCD, considering a threshold PI of 1.3 in conjunction with a FVd < 20 cm/sec for considering CBF changes potentially associated with high ICP, or for excluding it [9]. This remains in relation to TBI, where invasive monitoring is not available, and needs careful interpretation and comprehensive multi-modality assessment [25]. For those wishing to explore further, diastolic closing margin and critical closing pressure are vital to consider.

Internal carotid: A formal neurosonology examination of the bilateral carotids and vertebrals requires extensive and detailed assessment. For the purposes of BrainPOCUS in the critically ill, identification of the internal carotid and Doppler spectral analysis of such is principally performed to facilitate calculation of the Lindegaard ratio [27]. Identification of other pathologies, such as dissection flaps, vascular occlusion, or high-grade stenosis, could occur, but where there has been any question of such pathology from history, exam, or prior investigation, formal and detailed examination techniques (typically involving contrast CT or formal detailed ultrasound assessment) should be performed.

**Figure 10:**
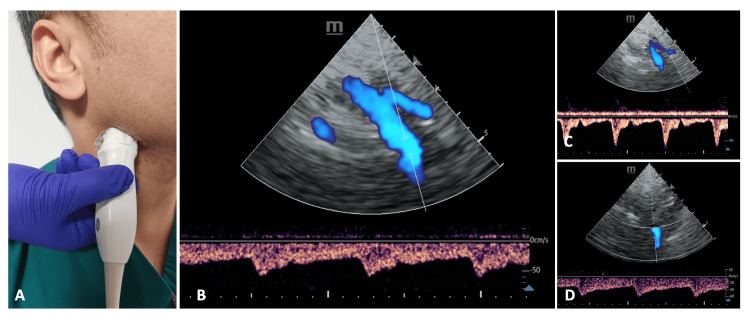
A simulated demonstration of the submandibular window assessment using the phased-array probe. A: Phased-array probe placement to image the submandibular window. B: Transcranial colour-coded triplex (TCCT) image with pulse-wave Doppler gate placed over the internal carotid artery. C: TCCT image with pulse-wave Doppler gate placed over the external carotid artery. D: TCCT image demonstrating how alignment can be adjusted. Of note, with inferior probe positioning and carotid flow cranially, blood flow is travelling away from the probe and is subsequently displayed in blue colour with a negative pulse-wave Doppler waveform. If desired, the Doppler default can be inverted, though measurements can be taken without requiring this step, with any recorded value easily converted to its equivalent positive value.

The probe most frequently used for carotid ultrasound assessment is the linear probe, with its longer, flat footprint and operating frequency of 5-10 MHz [23]. We will describe some basic linear approaches, though rapid spectral measurements can be obtained from the phased-array probe alone (see Figure 10). Most relevant to the critically ill patient, the presence of central venous catheters may hinder ultrasound probe placement and examination possibilities, in addition to the usual challenges that may be encountered in the general population (e.g. short neck or high bifurcation). 

**Figure 11:**
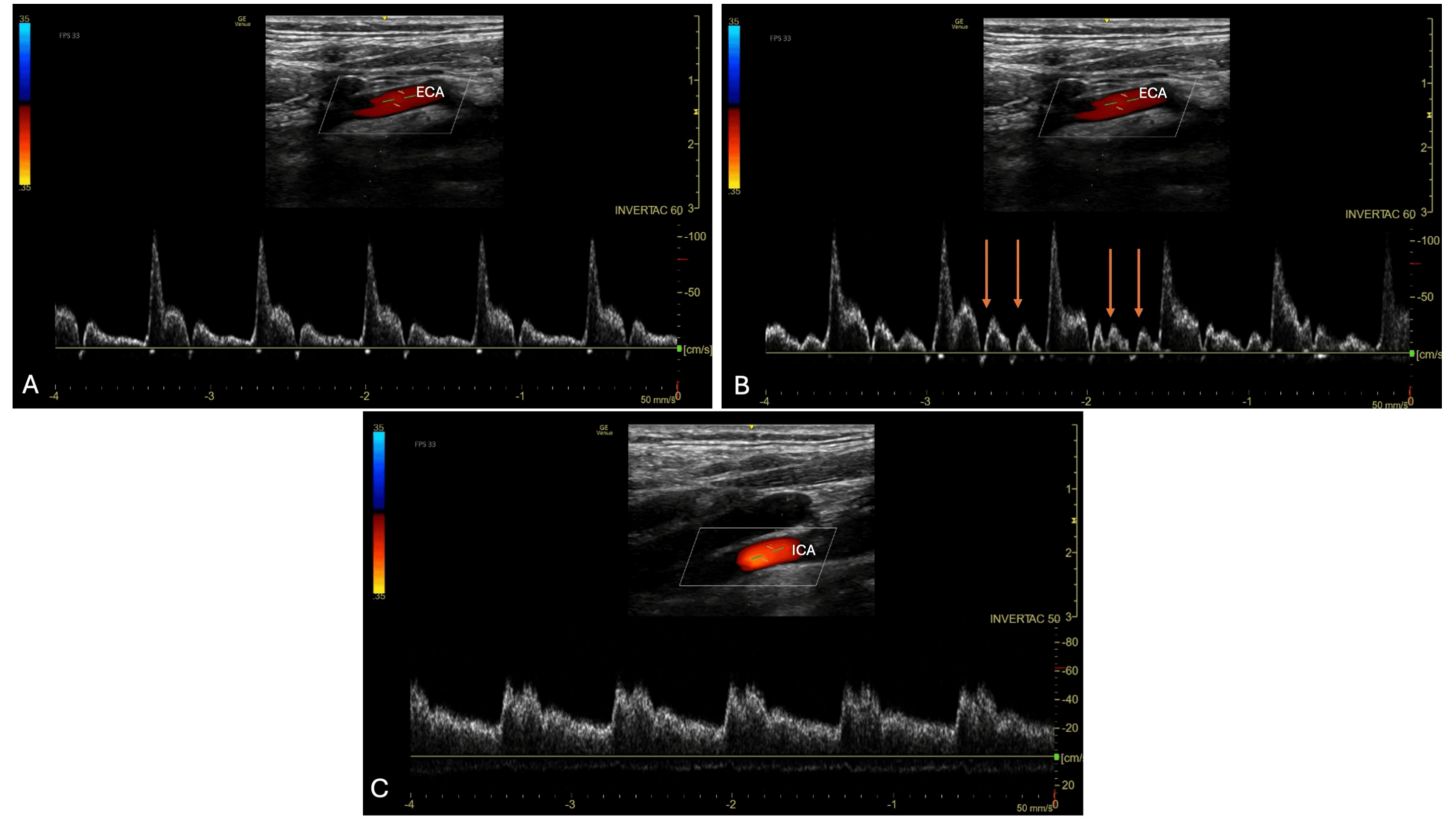
In-plane xarotid Doppler using a linear-array probe. A: External carotid artery with its higher resistance pattern. B: External carotid artery with transmitted ‘tapping’ oscillations demonstrated. C: Internal carotid artery with a lower resistance waveform pattern.

Scanning upward from the carotid bifurcation, it is crucial to distinguish between the external and internal carotid arteries - misidentifying the internal carotid artery (ICA) as the external carotid artery (ECA) can lead to incorrect calculations and inferences. For differentiation, the ICA is usually deeper and more posterior in position, lacks branch divisions, and normally has a low-resistance pattern with higher diastolic [14]. Typically, the most useful method to distinguish ICA from ECA is percussion oscillation assessment [6,14,23] (see Figure 11). Here, tapping percussion is performed over the ipsilateral temporal artery, and fluid percussion waves are visualised on Doppler spectra in the ECA but not the ICA. The distal ICA (depth: 40-60 mm) via the submandibular window should be used [6]. The Lindegaard ratio can be calculated by dividing the FV_mean_ of the ipsilateral MCA by the FV_mean_ of the ipsilateral extracranial ICA [27]. Again, there can be variation from the original description that requires consideration [27].

Optic nerve sheath diameter: Increasingly of value as part of a targeted BrainPOCUS exam, optic nerve sheath diameter (ONSD) assessment does require the use of a linear-array probe. Relevant disinfecting procedures of the probe prior to use, the use of water-soluble non-irritant gel, and a probe cover are important to mitigate risks. The probe is placed above the closed eyelid, with gel as usual used as a transmission medium, though here additional gel depth is used to avoid any pressure on the globe itself. Appropriate clinical concerns and justification are required. As mentioned previously, the ALARA principle should be followed, with specific recommendations from the FDA to ensure TI (itself time factored) less than or equal to 1 and MI less than or equal to 0.23 [11].

**Figure 12:**
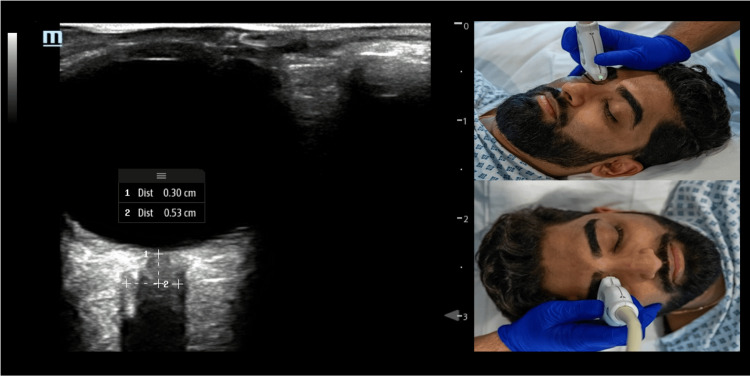
A simulated demonstration of the optic nerve sheath diameter (ONSD) assessment. Example of the ONSD image with ONSDint measured at 3 mm posterior to the retina, along with separate examples of simulated probe positioning.

The probe marker is directed medially (axial imaging), with tilting and depth adjustment to display a short segment of the optic nerve and accompanying sheath posterior to the globe. Meticulous care is required with the hand of the sonographer supported on the patient's forehead to avoid any orbital pressure transmission. Focal depth should be adjusted to the retina and the most proximal optic nerve. The width of the optic nerve sheath at 3 mm posterior to the retina, in line with the nerve axis, is measured. The internal ONSD (ONSDint) has been recommended for clinical practice standardization (see Figure 12) and should also be measured at right angles to the nerve axis [11]. There have been concerns around reproducibility and reliability; however, averaging is not recommended in the latest consensus document, and it risks excessively long scan duration [11].

ONSD for BrainPOCUS is contraindicated in the setting of ocular trauma. Other physiological considerations, such as underlying disease, specific clinical context, and parameters from other monitoring modalities, base of skull fracture, CSF leak, etc. should be considered [9]. Being such a delicate structure, assessment should be clinically justified and performed with utmost care to mitigate any risk. B-ICONIC is recommended when using ONSD, considering a threshold of 6 mm as a potential marker of intracranial hypertension or for excluding it [9].

## Discussion

Compared to other modalities of POCUS (e.g. cardiac), neurological or BrainPOCUS lags behind in regard to utilisation and training in the critical care setting [28]. BrainPOCUS can provide valuable physiological insights early in the course of neurological deterioration, facilitating more targeted decision-making in critically ill patients [9]. The physical principles of ultrasound underpin safe BrainPOCUS use in the ICU, including awareness of thermal and mechanical indices, balanced with clinical risks, concerns, and available alternatives. Technological developments with imaging, TCCD and TCCT, have been important for BrainPOCUS.

BrainPOCUS is an evolving technique finding its place somewhere between a comprehensive neurosonology investigation and a basic bedside physical examination. Whilst more advanced techniques exist, and certain notable areas are not included here (e.g. posterior circulation), obtaining a basic dataset that is rapid and reproducible is potentially most applicable to most critical care clinicians most of the time. Consensus skill recommendations on brain ultrasonography in critical care agree with this [7]. In critically ill patients that are sedated and possibly pharmacologically paralysed, BrainPOCUS is an accessible technique using equipment readily available to glean important clinical information not appreciable from clinical exam alone. Importantly, interpretation of data, appreciation of key pitfalls, clinical management strategies, and established accreditation and safeguarding processes associated with them are all of utmost importance to implementation at scale [29]. A recent systematic review has highlighted the current evidence gap between recommended applications and training delivery [30]. In addition, and comparable to all other POCUS modalities, infrastructural considerations relating to image storage, audit, and follow-up pathway development require careful local consideration.

When compared with CT and MRI, BrainPOCUS offers distinct advantages, with notable limitations. CT and CT angiography (CTA) investigation remain integral to detecting acute haemorrhage, large vessel occlusion (stroke), mass effect, and hydrocephalus, whilst MRI provides unrivalled resolution for structural pathology. BrainPOCUS cannot and does not replace these pathway-linked modalities, nor does it offer a comprehensive anatomical assessment. However, BrainPOCUS strengths are evident in situations where transport is unsafe, delayed, or impractical, and where real-time physiological data are required, typical in the critically ill cohort. BrainPOCUS delivers immediate physiological data that can be repeated at intervals to monitor and assess for clinical change. These considerations position BrainPOCUS as a useful complementary POCUS modality in critical care.

BrainPOCUS provides a practical, physiologically informative approach to bedside neurological assessment in critically ill patients. By combining structural and haemodynamic ultrasound techniques, clinicians can detect intracranial abnormalities, track dynamic changes, and support early diagnostic decisions when conventional examinations are limited. This article provides a structured methodology for performing a basic BrainPOCUS examination, designed to aid critical care clinicians in obtaining consistent datasets. Future directions for BrainPOCUS may include increased observational data collection, incorporation of BrainPOCUS within formal critical care training curricula, and interventional studies via approved interventional trials.

## Conclusions

BrainPOCUS can be considered a basic blend of multiple established neurosonology techniques, combined to provide a rapid but limited bedside structural and neurovascular assessment in any critically ill patient. BrainPOCUS is not to be confused with, or used in lieu of, a comprehensive neurosonology examination or any established and importantly pathway-linked detailed investigative method (e.g. CT/CTA imaging). However, BrainPOCUS provides a very limited and targeted dataset, a dataset that is especially relevant to intensivists. How these data are then interpreted or clinical management potentially adjusted, is beyond the remit of this current methods paper, and itself remains a key research priority.

## Figures and Tables

**Figure 13:**
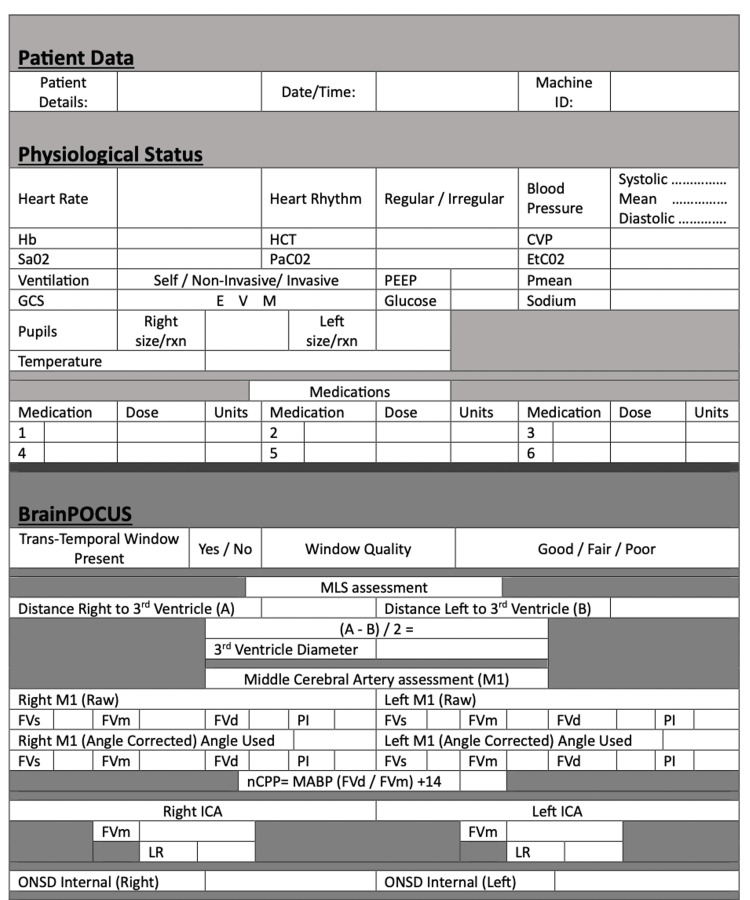
An example BrainPOCUS parameter proforma. BrainPOCUS: Brain point-of-care ultrasound
